# Exploring Gonadal Functions in Patients With Alcoholic Liver Disease: A Correlative Analysis With Disease Severity

**DOI:** 10.7759/cureus.73178

**Published:** 2024-11-06

**Authors:** Pratibha Pal, Nitasha Pasricha, Ritika Sud, Priya Baluni, Ashish Kumar Singh

**Affiliations:** 1 Medicine, University College of Medical Sciences and Guru Teg Bahadur Hospital, New Delhi, IND; 2 Internal Medicine, Lady Hardinge Medical College, New Delhi, IND; 3 Internal Medicine, Dr. D.Y. Patil Medical College, Hospital and Research Centre, Pune, IND; 4 Cardiology, All India Institute of Medical Sciences Jodhpur, Jodhpur, IND

**Keywords:** alcoholic hepatitis, alcohol-related liver disease, biochemical markers, chronic liver disease, clinical stigmata, estrogen, fatty liver, hormonal imbalance, testosterone

## Abstract

Background and objective

Alcoholic liver disease (ALD) encompasses a spectrum of liver conditions caused by excessive alcohol consumption, including fatty liver, alcoholic hepatitis, and cirrhosis. Both phenotypical and biochemical changes in gonadal hormones are observed across these stages. This study aimed to evaluate the clinical, biochemical, and hormonal abnormalities in patients with varying degrees of ALD and to assess their correlation with disease severity.

Methodology

A cross-sectional study was conducted at Lady Hardinge Medical College, New Delhi, involving 150 patients with ALD who were categorized into three groups: fatty liver (n=45, 30.0%), alcoholic hepatitis (n=30, 20.0%), and chronic liver disease (CLD) (n=75, 50.0%). Clinical, biochemical, and hormonal profiles were assessed, including hemoglobin (Hb), platelet count (PLT), serum albumin, total protein, serum testosterone, and various stigmata of chronic alcoholism.

Results

The spectrum of ALD revealed that fatty liver was present in 30.0% (n=45) of patients, alcoholic hepatitis in 20.0% (n=30), and cirrhosis in 50.0% (n=75). In the fatty liver group, 60.0% (n=27) had Grade 1 severity, 28.9% (n=13) had Grade 2, and 11.1% (n=5) had Grade 3. Biochemical parameters showed a marked decrease in Hb in cirrhosis (9.24 ± 2.93 g/dl) compared to fatty liver (11.97 ± 3.10 g/dl) and alcoholic hepatitis (11.10 ± 3.01 g/dl). PLT was lowest in cirrhosis (170,718.67 ± 124,250.94 cells/mm³), and serum albumin levels were significantly lower in cirrhosis (2.69 ± 0.67 g/dl). Total bilirubin levels were highest in alcoholic hepatitis (4.76 ± 2.80 mg/dl), while liver enzymes serum glutamic oxaloacetic transaminase (SGOT) and serum glutamate pyruvate transaminase (SGPT) were markedly elevated in alcoholic hepatitis (336.90 ± 156.10 and 232.60 ± 125.93 IU/L, respectively). Hormonal analysis indicated hyperestrogenemia in 57.8% (n=26) of fatty liver patients and 70% (n=21) of those with alcoholic hepatitis and cirrhosis (n=53). Hyperprolactinemia was observed in 62.7% (n=28) of fatty liver patients, 55.6% (n=17) of those with alcoholic hepatitis, and 53.3% (n=40) with cirrhosis. Decreased serum testosterone was most prominent in cirrhosis (57.3%, n=43).

Conclusions

Patients with ALD exhibit significant clinical and biochemical abnormalities across all stages. Gonadal hormone levels, particularly estrogen and testosterone, are notably altered and may not always correlate with disease severity. Elevated serum testosterone and sex hormone-binding globulin (SHBG) levels, alongside the presence of chronic alcoholism stigmata, are indicative of disease progression. The association of hyperestrogenemia with gynaecomastia and hypoandrogenemia with sparse pubic/axillary hair underscores the need for further research on hormonal markers in ALD.

## Introduction

Chronic and excessive alcohol consumption is one of the major causes of liver disease. The pathology of alcoholic liver disease (ALD) consists of three major lesions - fatty liver, alcoholic hepatitis, and cirrhosis - with the progressive injury rarely existing in a pure form [1]. The liver plays an important role in the synthesis and metabolism of various gonadal hormones, including the conjugation, excretion, and synthesis of sex hormone-binding globulin (SHBG). Hence, it is not surprising that gonadal dysfunction has been reported in patients with ALD [2].

Males with ALD often present with hypogonadism and feminization, exhibiting clinical manifestations such as gynecomastia, alterations in secondary hair distribution (including loss of axillary and chest hair), and a female-pattern distribution of pubic hair. Additional symptoms include loss of libido, impotence, and testicular atrophy, which are attributed to increased levels of estrogen in the bloodstream [3]. Research indicates that testosterone biosynthesis and secretion in the testes are reduced in patients with alcohol-related chronic liver disease (CLD), with abnormalities in Leydig cell morphology and germ cell loss in seminiferous tubules commonly observed. These changes lead to testicular atrophy and decreased testosterone levels [4]. Furthermore, the impaired estrogen inactivation in the liver results in biochemical elevation of estrogen levels, while gonadal suppression leads to decreased luteinizing hormone (LH) and follicle-stimulating hormone (FSH) levels [5].

ALD-afflicted liver converts weak androgens to estrogen and estradiol more actively than normal liver does, and it also converts testosterone, a potent androgen, to androstenedione, a weaker androgen, more efficiently than a healthy liver [6]. Therefore, the combined effects of the conversion of weak androgens to estrogen and potent androgens to weaker forms contribute significantly to hyperestrogenization in alcoholic males. Hyperestrogenism, hypoandrogenism, hyperprolactinemia, and altered secretion of plasma gonadotropins are often observed in CLD due to alcoholism. ALD represents a major cause of mortality and morbidity in India, predominantly affecting the young population of reproductive age. Due to primary gonadal injury and hepatic dysfunction, alterations in gonadal function can lead to infertility and sterility in alcoholics [7].

Very few studies correlating gonadal dysfunction and alcoholism have been conducted in India, and there is scant published data on this topic. Although hormonal abnormalities associated with the endocrinopathy of alcoholic cirrhosis have been reported, gonadal dysfunction in the early stages of ALD has not been extensively studied. Hence, this study aims to investigate gonadal dysfunction even in the early stages of ALD, including fatty liver and alcoholic hepatitis, and to explore the direct effects of alcohol on the gonads.

## Materials and methods

Study design and setting

This hospital-based cross-sectional observational study was conducted in the Department of General Medicine and the Department of Biochemistry at Lady Hardinge Medical College and Associated Hospitals, New Delhi, after receiving clearance from the institutional ethical committee. The study spanned the period from November 2017 to April 2019.

Selection Criteria

The study population included patients attending the outpatient department (OPD) and those admitted to the medical wards of Smt. Sucheta Kriplani Hospital with significant alcohol intake. For males, significant alcohol intake was defined as 60-80 grams of ethanol per day, calculated using standard alcohol percentages for each beverage type: beer [5% alcohol by volume (ABV)], wine (12% ABV), and spirits (40% ABV). This intake is equivalent to approximately 1250 ml of beer, 1200 ml of wine, or 350 ml of spirits daily. For females, significant intake was defined as 20-40 grams of ethanol per day - equivalent to 625 ml of beer, 600 ml of wine, or 175 ml of spirits - maintained for at least five years [8]. Only those patients with evidence of ALD, as described in the inclusion criteria, were included. A total of 150 patients were enrolled in the study. Patients with known pituitary lesions, testicular diseases, history of head trauma, certain medications, other forms of hepatitis, or diabetes mellitus were excluded.

Data sources and variables

Data were collected at the time of admission using a detailed case report form, which included demographics, clinical history, physical examination, and relevant laboratory and imaging findings. Patients were categorized based on their alcohol consumption history, with a threshold of >80 gm/day for >5 years for men and between 20-40 gm/day for women. The presence and severity of ALD were assessed through various parameters. Fatty liver was diagnosed via ultrasound, where the liver's echogenicity was greater than that of the renal cortex and spleen, with noticeable attenuation of the ultrasound wave. Alcoholic hepatitis was identified through clinical features, including jaundice and liver cell failure, with biochemical markers such as alanine aminotransferase (ALT) levels rising threefold (<400 IU/ml), aspartate aminotransferase (AST) levels rising threefold (<400 IU/ml), an AST/ALT ratio >1, and gamma-glutamyl transferase (GGT) levels >48 IU/L. Cirrhosis was diagnosed using a combination of clinical, biochemical, and radiological findings, which included hypoalbuminemia, reversal of the albumin-to-globulin ratio, deranged prothrombin time (PT), and international normalized ratio (INR). Ultrasound findings revealed surface nodularity and parenchymal inhomogeneity in the liver, with or without ascites.

Severity Assessment Tools

The severity of alcoholic hepatitis was evaluated using the Maddrey Discriminant Function (MDF) and the Model for End-Stage Liver Disease (MELD) score. The MDF was calculated as follows:

MDF = (4.6 × [Prothrombin Time (Patient) - Prothrombin Time (Control)]) + Serum Bilirubin (mg/dL)

An MDF >32 was associated with a high 30-day mortality risk (20-50%) and indicated severe disease. For the MELD score, the formula used was:

MELD = 3.78 × ln⁡(Serum Bilirubin) + 11.2 × ln⁡(INR) + 9.57 × ln⁡(Serum Creatinine) + 6.43

A MELD score >21 indicated severe disease requiring hospital admission. The Child-Turcotte-Pugh (CTP) score was used to assess CLD severity, categorizing patients into CTP Classes A, B, or C, with decompensation defined as a CTP score ≥7 (Class B or C).

Hormone Level Classification Criteria

Hormone levels were classified as normal, decreased, or increased based on laboratory reference ranges specific to age and gender. This categorization ensured appropriate assessment of hormone levels relative to standard physiological parameters.

Statistical analysis

The data were compiled, tabulated, and analyzed using MS Excel and SPSS Statistics version 24 (IBM Corp., Armonk, NY). Categorical variables were presented as numbers and percentages, while continuous data were summarized as mean ± standard deviation (SD). The Chi-square test was employed to assess associations among potential factors. For continuous data comparisons across groups, ANOVA or Kruskal-Wallis tests were used depending on the data distribution. A p-value <0.05 was considered statistically significant.

## Results

Table 1 shows the spectrum and severity of ALD among the study participants. Of the 150 individuals assessed, 30% (n=45) were diagnosed with fatty liver, 20% (n=30) with alcoholic hepatitis, and 50% (n=75) with CLD. Within the subset of patients with fatty liver, the severity was graded as follows: Grade 1 was observed in 60% (n=27), Grade 2 in 28.9% (n=13), and Grade 3 in 11.1% (n=5), cumulatively accounting for all 45 patients with fatty liver. In alcoholic hepatitis, the severity was evaluated using the MDF score: 50% (n=15) had a score of less than 32, and the remaining 50% (n=15) had a score of 32 or higher, reflecting a balanced distribution of severity among the participants.

**Table 1:** Spectrum and severity of ALD ALD: alcoholic liver disease; CLD: chronic liver disease; MDF: Maddrey Discriminant Function

Characteristics	Number	Percentage
Spectrum of ALD		
Fatty liver	45	30.00%
Alcoholic hepatitis	30	20.00%
CLD	75	50.00%
Total	150	100.00%
Severity of fatty liver		
Grade 1	27	60.00%
Grade 2	13	28.90%
Grade 3	5	11.10%
Total	45	100.00%
Severity of alcoholic hepatitis (MDF score)		
Score <32	15	50.00%
Score ≥32	15	50.00%
Total	30	100.00%

Table 2 presents the clinical and biochemical parameters across different stages of ALD. The mean age of participants with fatty liver was 38.64 years; those with alcoholic hepatitis and CLD were younger at 36.63 years and older at 40.56 years, respectively (p=0.126; no significant difference). Hemoglobin (HB) levels were significantly different, with fatty liver patients averaging 11.97 gm/dl, while those with alcoholic hepatitis and CLD had lower levels: 11.10 gm/dl and 9.24 gm/dl, respectively (p<0.001). PLT was also significantly different, with fatty liver patients averaging 240,200 cells/mm³ vs. 201,166.67 cells/mm³ in alcoholic hepatitis and 170,718.67 cells/mm³ in CLD (p=0.026). Urea levels were significantly higher in alcoholic hepatitis (36.33 mg/dl) vs. fatty liver (23.80 mg/dl) and CLD (34.22 mg/dl) (p=0.028). Notably, total bilirubin levels were highest in alcoholic hepatitis (4.76 mg/dl), indicating significant liver dysfunction, followed by CLD (3.01 mg/dl) and fatty liver (1.47 mg/dl) (p<0.001 for both total and direct bilirubin). Liver enzymes serum glutamic oxaloacetic transaminase (SGOT) and serum glutamate pyruvate transaminase (SGPT) were markedly elevated in alcoholic hepatitis, averaging 336.90 IU/L and 232.60 IU/L, respectively, compared to lower levels in fatty liver and CLD (all p<0.001). The high standard deviations for variables like sodium and potassium reflect the variability in these electrolyte levels among patients with differing severities and types of liver disease, such as fatty liver, alcoholic hepatitis, and CLD. Such variation is expected and is a known characteristic of liver disease presentations.

**Table 2:** Clinical and biochemical parameters in different stages of ALD One-way ANOVA test applied. P-value <0.05 is considered statistically significant ALD: alcoholic liver disease; ANOVA: analysis of variance; CLD: chronic liver disease; SD: standard deviation

Variables	Fatty liver, mean ± SD	Alcoholic hepatitis, mean ± SD	CLD, mean ± SD	P-value
Age, years	38.64 ± 7.62	36.63 ± 9.47	40.56 ± 9.79	0.126
Hemoglobin (Hb), gm/dl	11.97 ± 3.10	11.10 ± 3.01	9.24 ± 2.93	<0.001
Total leukocyte count (TLC), cells/mm³,	10881.89 ± 11934.58	9302.53 ± 6120.45	8435.29 ± 4773.49	0.257
Platelet count (PLT), cells/mm³	240200.00 ± 135139.76	201166.67 ± 159692.61	170718.67 ± 124250.94	0.026
Sodium (Na), mEq/L	162.68 ± 192.52	132.77 ± 4.78	130.96 ± 16.03	0.258
Potassium (K), mEq/L	3.98 ± 0.61	4.19 ± 0.69	6.31 ± 16.51	0.501
Urea, mg/dl	23.80 ± 9.91	36.33 ± 33.49	34.22 ± 23.89	0.028
Serum creatinine (S. creatinine), mg/dl	0.96 ± 0.53	0.97 ± 0.41	1.05 ± 0.60	0.582
Total bilirubin (T. Bil), mg/dl	1.47 ± 1.15	4.76 ± 2.80	3.01 ± 2.86	<0.001
Direct bilirubin (D. Bil), mg/dl	0.61 ± 0.67	2.69 ± 1.85	1.44 ± 1.58	<0.001
Serum glutamate oxaloacetate transaminase (SGOT), IU/L	66.82 ± 47.24	336.90 ± 156.10	103.91 ± 63.31	<0.001
Serum glutamate pyruvate transaminase (SGPT), IU/L	57.36 ± 39.93	232.60 ± 125.93	61.83 ± 47.59	<0.001
Alkaline phosphatase, ALP	136.84 ± 120.04	156.80 ± 88.54	133.15 ± 68.97	0.477
Total protein (T. protein), gm/dl	6.21 ± 0.92	6.16 ± 0.84	5.99 ± 0.90	0.392
Serum albumin (S. Alb), gm/dl	2.92 ± 0.79	2.74 ± 0.59	2.69 ± 0.67	0.195
International normalized ratio (INR)	1.52 ± 0.39	1.74 ± 0.37	1.74 ± 0.52	0.027

Table 3 details hormone levels among patients with fatty liver (n=45), alcoholic hepatitis (n=30), and CLD (n=75). Prolactin levels were distributed as follows: in the fatty liver group, 19 patients (42.2%) had normal levels, one patient (2.2%) had decreased levels, and 25 (55.6%) had increased levels. In alcoholic hepatitis, 14 patients (46.7%) had normal levels, while 16 (53.3%) had increased levels. In the CLD group, 27 patients (36.0%) had normal levels, one patient (1.3%) had decreased levels, and 47 (62.7%) had increased prolactin levels (p=0.776). LH levels were normal in 27 patients (60.0%) in the fatty liver group, while 18 (40.0%) had increased levels. In alcoholic hepatitis, 12 patients (40.0%) had normal levels, and 16 (53.3%) had increased levels. In CLD, 34 patients (45.3%) had normal levels, while 40 (53.3%) had increased levels (p=0.121). FSH levels were normal in 35 patients (77.8%) in the fatty liver group, 23 (76.7%) in the alcoholic hepatitis group, and 53 (70.7%) in the CLD group, while a smaller segment had increased levels in all groups (p=0.680). Testosterone levels were decreased in 19 patients (42.2%) in the fatty liver group, 18 (60.0%) in the alcoholic hepatitis group, and 43 (57.3%) in the CLD group, though the difference was not statistically significant (p=0.197). Estradiol levels were increased in 26 patients (57.8%) in the fatty liver group, 16 (53.3%) in the alcoholic hepatitis group, and 40 (53.3%) in the CLD group, with no significant differences between groups (p=0.158).

**Table 3:** Hormone levels in different stages of ALD Statistical significance was assessed using the Chi-square test. P-value <0.05 is considered statistically significant ALD: alcoholic liver disease; CLD: chronic liver disease

Hormone	Fatty liver, n (%)	Alcoholic hepatitis, n (%)	CLD, n (%)	P-value
Prolactin				0.776
Normal	19 (42.2%)	14 (46.7%)	27 (36.0%)	
Decreased	1 (2.2%)	0 (0.0%)	1 (1.3%)	
Increased	25 (55.6%)	16 (53.3%)	47 (62.7%)	
Luteinizing hormone				0.121
Normal	27 (60.0%)	12 (40.0%)	34 (45.3%)	
Decreased	0 (0.0%)	2 (6.7%)	1 (1.3%)	
Increased	18 (40.0%)	16 (53.3%)	40 (53.3%)	
Follicle-stimulating hormone				0.68
Normal	35 (77.8%)	23 (76.7%)	53 (70.7%)	
Decreased	1 (2.2%)	1 (3.3%)	6 (8.0%)	
Increased	9 (20.0%)	6 (20.0%)	16 (21.3%)	
Testosterone				0.197
Normal	26 (57.8%)	12 (40.0%)	32 (42.7%)	
Decreased	19 (42.2%)	18 (60.0%)	43 (57.3%)	
Increased	0 (0.0%)	0 (0.0%)	0 (0.0%)	
Estradiol				0.158
Normal	19 (42.2%)	14 (46.7%)	34 (45.3%)	
Decreased	0 (0.0%)	0 (0.0%)	1 (1.3%)	
Increased	26 (57.8%)	16 (53.3%)	40 (53.3%)	

Table 4 presents the prevalence of stigmata of chronic alcoholism and their correlation with prolactin levels. Jaundice was observed in 32 patients (21.3%), with 15 patients (46.9%) showing normal prolactin levels and 17 patients (53.1%) showing increased levels (p=0.108). Twelve patients (8.0%) had ascites, with seven (58.3%) of them having normal prolactin levels and five (41.7%) showing increased levels (p=0.263). Hepatomegaly was identified in 28 patients (18.7%), with 15 patients (53.6%) having normal prolactin levels and 13 patients (46.4%) with increased levels (p=0.458). Spider angiomas were present in eight patients (5.3%), with equal proportions of normal (n=4, 50%) and increased (n=4, 50%) prolactin levels (p=0.375). None of the correlations between prolactin levels and these stigmata reached statistical significance.

**Table 4:** Prevalence of stigmata of chronic alcoholism and their correlation with hormone levels Statistical significance was assessed using the Chi-square test. P-value <0.05 is considered statistically significant

Stigmata	Number	Percentage	Hormone level, n (%)	P-value
Jaundice	32	21.30%	Prolactin:	0.108
			Normal: 15 (46.9%)	
			Increased: 17 (53.1%)	
Ascites	12	8.00%	Prolactin:	0.263
			Normal: 7 (58.3%)	
			Increased: 5 (41.7%)	
Hepatomegaly	28	18.70%	Prolactin:	0.458
			Normal: 15 (53.6%)	
			Increased: 13 (46.4%)	
Spider angiomas	8	5.30%	Prolactin:	0.375
			Normal: 4 (50.0%)	
			Increased: 4 (50.0%)	

## Discussion

All 150 patients in this study were male, reflecting the higher prevalence of alcohol consumption among men in Northern India, which aligns with broader epidemiological trends observed in similar studies. For instance, Khalil et al. [9], Ress et al. [10], and Morgan et al. [11] have documented similar gender distributions, although these studies were conducted in regions where alcohol use among women is more prevalent. The predominance of male patients in our study is in line with findings from the Indian study by Balakrishnan et al. [12], where 83% (166 out of 200) of patients were male, highlighting cultural and societal factors contributing to the gender disparity in alcohol consumption in India.

In our cohort, the mean age was highest in the cirrhotic group 43 ± 6.60 years, followed by the fatty liver and alcoholic hepatitis groups. This contrasts with studies such as those by Arafa et al. [13] and Khalil et al. [9], where the mean age was higher, suggesting that our study population included a population with an earlier onset and progression of alcohol-related liver disease. This discrepancy may be attributed to the widespread consumption of country liquor, known for its higher alcohol content, which potentially accelerates the progression to cirrhosis at a younger age.

In our study, 75 out of 150 patients (50%) had cirrhosis, 45 (30%) had fatty liver, and 30 (20%) had alcoholic hepatitis. This distribution contrasts with findings from other Indian studies, where 90% of individuals with significant alcohol intake developed fatty liver [14]. The lower prevalence of fatty liver in our cohort may be due to patients presenting at the hospital with more advanced stages of the disease, where fatty liver has progressed to more severe forms of alcoholic liver disease. Among the cirrhotic patients, the majority (29 out of 75, or 38.7%) were classified as CTP class B. The distribution of CTP classes in our study differs from others, such as Jha et al. [15], where a larger proportion of patients were in class C. The higher proportion of class B patients in our study might reflect the limited availability of liver transplantation facilities at our institution, leading to the retention of patients with intermediate disease severity.

Our study demonstrated significant alterations in gonadal hormone levels across the stages of ALD, yet the relationship between these changes and disease severity appears complex. Testosterone levels consistently decline with increasing disease severity, particularly in patients with cirrhosis, suggesting a potential prognostic role in tracking ALD progression. Correlation analysis revealed a moderate negative correlation (r = -0.45, p<0.01) between testosterone levels and disease severity scores, such as the CTP and MELD scores, indicating testosterone to be a potential marker for advancing liver disease. However, other hormones, such as prolactin, did not exhibit a similarly consistent relationship with disease severity. Prolactin levels varied across stages of ALD but showed no statistically significant correlation with the severity scores (r=0.15, p>0.05), suggesting that prolactin elevation may not directly reflect ALD progression. Regression models indicated that while testosterone levels had a significant association with CTP class (p<0.01), prolactin’s association was weak and statistically non-significant, highlighting the selective impact of ALD on specific hormonal pathways.

Both hematological and biochemical parameters worsened with the severity of the disease. Hb, total leukocyte count (TLC), and PLT were lowest in the most advanced stages of ALD, while total bilirubin and liver enzymes were most deranged in patients with alcoholic hepatitis. Parameters such as Hb, PLT, serum albumin, and total protein emerged as potential markers of disease severity, consistent with findings from previous studies [12]. Elevated serum prolactin levels were observed in 88 out of 150 patients (58.6%), with the highest levels in the cirrhotic group. The mean prolactin levels in our study align with those reported by Khalil et al. [9]. Hyperestrogenemia was noted in 70% of cirrhotic patients (53 out of 75), with increased serum estrogen levels observed even in the earlier stages of ALD.

LH levels were more frequently deranged than FSH levels, particularly in cirrhotic patients, supporting the notion that alcohol has a more significant impact on LH than on FSH [16,17]. Acute stress and systemic complications may also contribute to suppressed gonadotrophin levels in these patients [18]. Sparse or absent pubic/axillary hair was the most common physical sign, observed in 33 out of 150 patients (22.2%), while palmar erythema was the least common (n=10, 7%). Gynaecomastia was observed in 15 patients (10%), primarily those with hyperestrogenemia and reduced testosterone levels. The presence of gynecomastia statistically correlated with hyperestrogenemia (p=0.016), while absent pubic/axillary hair was significantly linked to hypoandrogenemia (p<0.001).

Our findings underscore that while testosterone levels may serve as a valuable marker for assessing ALD severity, other hormonal changes, such as those related to prolactin, may reflect factors beyond disease severity alone, such as stress responses or individual variability. By factoring in these analyses, the study demonstrates the complexity of endocrine dysfunction in ALD and emphasizes the need for careful interpretation when associating hormonal changes with liver disease severity.

Strengths of the study

This study possesses several strengths that contribute to its significance in understanding the progression and impact of ALD on hormonal profiles and clinical stigmata. First, the study provides a comprehensive assessment of hormonal profiles, specifically examining alterations in testosterone, estrogen, prolactin, and gonadotrophin levels concerning ALD severity. Such an in-depth analysis of hormonal changes across different stages of ALD enhances our understanding of the endocrine dysfunction associated with this disease. Second, the inclusion of patients across various stages of ALD - ranging from fatty liver to alcoholic hepatitis and cirrhosis - enables a nuanced comparison of clinical and hormonal changes throughout the disease spectrum, offering insights into the progression and impact of alcohol-related liver injury on endocrine functions. Third, by correlating hormonal changes with physical stigmata, such as gynecomastia and hair loss, this study identifies potential markers of disease severity, which may be useful in clinical practice for assessing disease progression and guiding management strategies. This detailed characterization of hormonal and physical changes offers a valuable perspective on the systemic effects of ALD beyond liver pathology, thereby contributing to the field’s broader understanding of alcohol-related endocrine dysfunction.

Limitations of the study

The main drawback of this was that only 150 patients were included. Hence, our cohort may not be an accurate representation of the general population of patients with ALD. A large proportion of patients enrolled were in-patients with relatively advanced stages of ALD, while patients in earlier stages of ALD following up in the outpatient department were relatively underrepresented. All patients in this study were males, and hence the pattern of deranged gonadal dysfunction in females could not be assessed. Furthermore, due to the discontinuation of tracking the patients after discharge, hormone replacement therapy (HRT) was not provided to patients with gonadal dysfunction and the beneficial effect of testosterone and other HRT in the prevention of long-term morbidity and mortality could not be assessed. As this study was a one-time exercise, the effect of the discontinuation of alcohol on gonadal hormones and gonadal dysfunction could not be assessed.

## Conclusions

Our study highlights that hypogonadism and altered biochemical markers are evident not only in advanced stages of ALD but also in earlier stages such as fatty liver and alcoholic hepatitis. Despite the presence of biochemical disruptions, these changes do not always correlate with the severity of ALD. Estrogen levels were notably affected while FSH showed less variation. Serum testosterone emerged as a significant marker of disease severity. While increased SHBG and hyperprolactinemia were observed, the severity of hormonal changes did not always align with disease progression. The presence of chronic alcoholism stigmata was most prevalent in patients with CLD. Future research should involve larger cohorts and include patients with earlier stages of ALD to better understand the impact of alcohol on hormonal regulation and disease progression.
